# Prenatal diagnosis and postnatal verification in fetuses with total anomalous pulmonary venous connection

**DOI:** 10.3389/fped.2023.1206032

**Published:** 2023-06-07

**Authors:** Xiaoying Xue, Qiumei Wu, Mingtao Xiong, Wen Ling, Shan Guo, Hong Ma, Biying Huang, Min Liu, Xiuqing Qiu, Zongjie Weng

**Affiliations:** ^1^ Department of Medical Ultrasonics, Fujian Maternity and Child Health Hospital, College of Clinical Medicine for Obstetrics & Gynecology and Pediatrics, Fujian Medical University, Fuzhou, China; ^2^ Department of Ultrasound, International Peace Maternity & Child Health Hospital, Shanghai Jiao Tong University School of Medicine, Shanghai, China; ^3^ Department of Pathology, Fujian Maternity and Child Health Hospital, College of Clinical Medicine for Obstetrics & Gynecology and Pediatrics, Fujian Medical University, Fuzhou, China; ^4^ Department of Obstetrics & Gynecology, Fujian Maternity and Child Health Hospital, College of Clinical Medicine for Obstetrics & Gynecology and Pediatrics, Fujian Medical University, Fuzhou, China

**Keywords:** total anomalous pulmonary venous connection, four-step approach, postnatal verification, ultrasound, prenatal diagnosis

## Abstract

**Objective:**

To systematically verify the accuracy of a four-step prenatal ultrasonography in diagnosing fetal total anomalous pulmonary venous connection (TAPVC).

**Methods:**

A total of 62 TAPVC fetuses received prenatal ultrasonography and were confirmed by postnatal echocardiography, surgery, or postabortion autopsy. The suspected TAPVC fetuses were further screened by a four-step prenatal ultrasonography for TAPVC classification, pulmonary venous obstruction, and the associated malformations, and followed postpartum. The sonographic features, clinical data, and prognosis of the TAPVC fetuses were retrospectively analyzed.

**Results:**

Of the 62 TAPVC fetuses, supracardiac TAPVC was found in 20 cases, intracardiac TAPVC in 12, infracardiac TAPVC in 21, and mixed TAPVC in 9. A total of 30 cases with right atrium isomerism were correctly diagnosed. Of the 11 cases with other intracardiac and extracardiac malformations, 1 case was missed to be diagnosed. Of the 21 isolated TAPVC cases, 6 were missed prenatally and 1 case was prenatally diagnosed as intracardiac and postnatally proved to be mixed (intracardiac type + supracardiac type) by echocardiography. Of the 13 TAPVC live births, 4 infants died in the neonatal period without operation. Of the nine infants undergoing the operation, five recuperated and survived; one survived but had complications with superior vena cava obstruction and collateral circulation formation, and three died postoperatively.

**Conclusion:**

The four-step prenatal ultrasound procedure can comprehensively and systematically evaluate fetal TAPVC, detailing the classification, potential obstruction, and associated malformations. It provides substantial support for subsequent prenatal counseling and neonatal assessment. The retrospective analysis also reveals that isolated TAPVC is more prone to be missed in diagnosis.

## Introduction

Total anomalous pulmonary venous connection (TAPVC) is a relatively rare cyanotic congenital heart disease, accounting for approximately 0.5%–2% of postnatal congenital heart diseases, in which all pulmonary veins (PVs) are not connected to the left atrium (LA) but directly or indirectly to the right atrium (RA) (1). According to different drainage pathways, TAPVC can be divided into four types: supracardiac, intracardiac, infracardiac, and mixed, with supracardiac type being the most common malformation and accounting for about 43% of all cases (2). TAPVC can occur alone or can accompany other deformities, and the most common comorbidity is right atrium isomerism (RAI) (3). Postnatally, TAPVC neonates often suffer from severe expiratory dyspnea, cyanosis, heart failure, etc. In case of pulmonary vein obstruction (PVO) complication, prompt emergency surgery is required; otherwise, most would die in the neonatal period. The prognosis of TAPVC is related to the specific subtypes, the presence of PVO, and associated malformations (4, 5). Therefore, an accurate prenatal diagnosis of TAPVC is of great significance for neonatal prognosis.

To date, the accuracy of TAPVC diagnosis varies significantly across different countries, regions, and institutions (3, 6). Some new ultrasound indicators or methods have been proposed to improve the prenatal diagnosis of TAPVC. For instances, post-LA space has been suggested as a potential indicator for prenatal diagnosis of TAPVC in many studies (7, 8). A four-dimensional spatiotemporal image correlation has been used to diagnose fetal TAPVC (9). Additionally, fetal PV variability index and vertical vein (VV) flow velocity have shown to predict postpartum PVO (10). Artificial intelligence can also be used to assist in diagnosing TAPVC (11). The routine prenatal screening has also identified pulmonary venous connections on a four-chamber view (12). Available literature demonstrates that prenatal screening, color Doppler, and spectral Doppler can identify 80% of suspected TAPVC, 34% of PVO, 59% of ventricular imbalance, 58% of increased LA posterior space, and 59% of VV cases (3). However, much remains to be desired with regard to a systemic assessment of TAPVC.

Given that the detection of PVO and the identification of its drainage pathway, an indispensable procedure of ultrasound diagnosis, are related to the prognosis of neonates with TAPVC, we proposed a four-step ultrasound approach to diagnose fetal TAPVC systematically, which demonstrated a favorable accuracy in prenatal diagnosis of TAPVC. The proposed method can provide a reliable support for prenatal consultation and clinical decision-making.

## Materials and methods

A total of 62 TAPVC fetuses underwent prenatal ultrasound examination in the Fujian Maternal and Child Health Hospital between June 2012 and June 2022, and were confirmed by postnatal echocardiography, surgical procedures, or postabortion autopsy. The clinical data and imaging findings of these TAPVC cases were retrospectively analyzed. Inclusive criteria were as follows: the fetuses with TAPVC were examined by systematic ultrasonography and verified by the above-mentioned means in our hospital. The exclusion criteria were as follows: the fetuses with TAPVC that were not verified postnatally or pathologically; RAI cases that indicated the entry of PV into the atrium; or cases that reported incomplete clinical data or no follow-up. The study protocol was reviewed and approved by the Ethics Committee of the Fujian Maternal and Child Health Hospital. Informed consent was obtained from all pregnant women.

### Prenatal ultrasonography

For prenatal ultrasonography, GE Voluson E8 and E10 high-resolution color Doppler ultrasound diagnostic instruments were employed, with the frequency of the convex array probe set at 2–9 MHZ, and the conditions of middle and late pregnancy and fetal heart examination were selected. The fetuses were systematically evaluated following the ISUOG guidelines for the prenatal ultrasonography (13, 14). TAPVC was suspected with the presence of any of the following signs: a small LA, an increased distance from the LA to the descending aorta, a smooth posterior wall of the LA, unobservable orifices of the PV, evident extra vessels and centrifugal venous flow in some sections of the heart, and an abnormal dilated vein [e.g., superior vena cava (SVC), innominate vein (INN), azygos vein (AZV), inferior vena cava (IVC), or coronary sinus (CS)]. The suspected TAPVC cases were further assessed by a four-step ultrasound procedure: (1) no connection was observed between all the PVs and the LA, in which the orifices of the PV were detected by two-dimensional echocardiography and color Doppler flow imaging, and the waveform of the PV was examined by spectral Doppler imaging; (2) the presence of the common pulmonary vein (CPV) was detected behind and beside the LA, and the drainage route of PV to the systematic vein via the CPV and VV was traced, on which the classification of TAPVC was based; (3) the presence and location of obstruction of the pulmonary venous drainage pathway were assessed by color Doppler and spectral Doppler imaging, in which color Doppler flow imaging may indicate turbulent blood flow at the site of obstruction, with a maximum flow velocity above 50 cm/s (15); (4) the fetal cardiac structures and extracardiac systems were examined in detail to determine the presence of intracardiac and extracardiac malformations.

### Postnatal verification

All the prenatally diagnosed fetal TAPVC cases underwent a multidisciplinary consultation. For cases of termination of pregnancy (TOP), autopsy was performed, with informed consent obtained from the parents, by either local anatomy or combined casting of the thoracic and abdominal organs, in which all specimens were archived, photographed, videotaped, and recorded. For live births, echocardiography or other imaging examinations were performed postnatally, in which Phillips EPIQ 7C was employed for neonatal echocardiography, with the frequency of phased array probes set at 3–8 MHz. These live births all received follow-ups.

### Statistical analysis

The data were analyzed with SPSS 25.0. The quantitative data displayed a normal distribution with homogeneous variance and were expressed as mean ± SD. For qualitative data, frequency (*N*) and percentage (%) were used. Comparisons were analyzed by the chi-square test with continuity correction. A value of *P* <0.05 was considered statistically significant.

## Results

### Data overview

**Figure 1:**
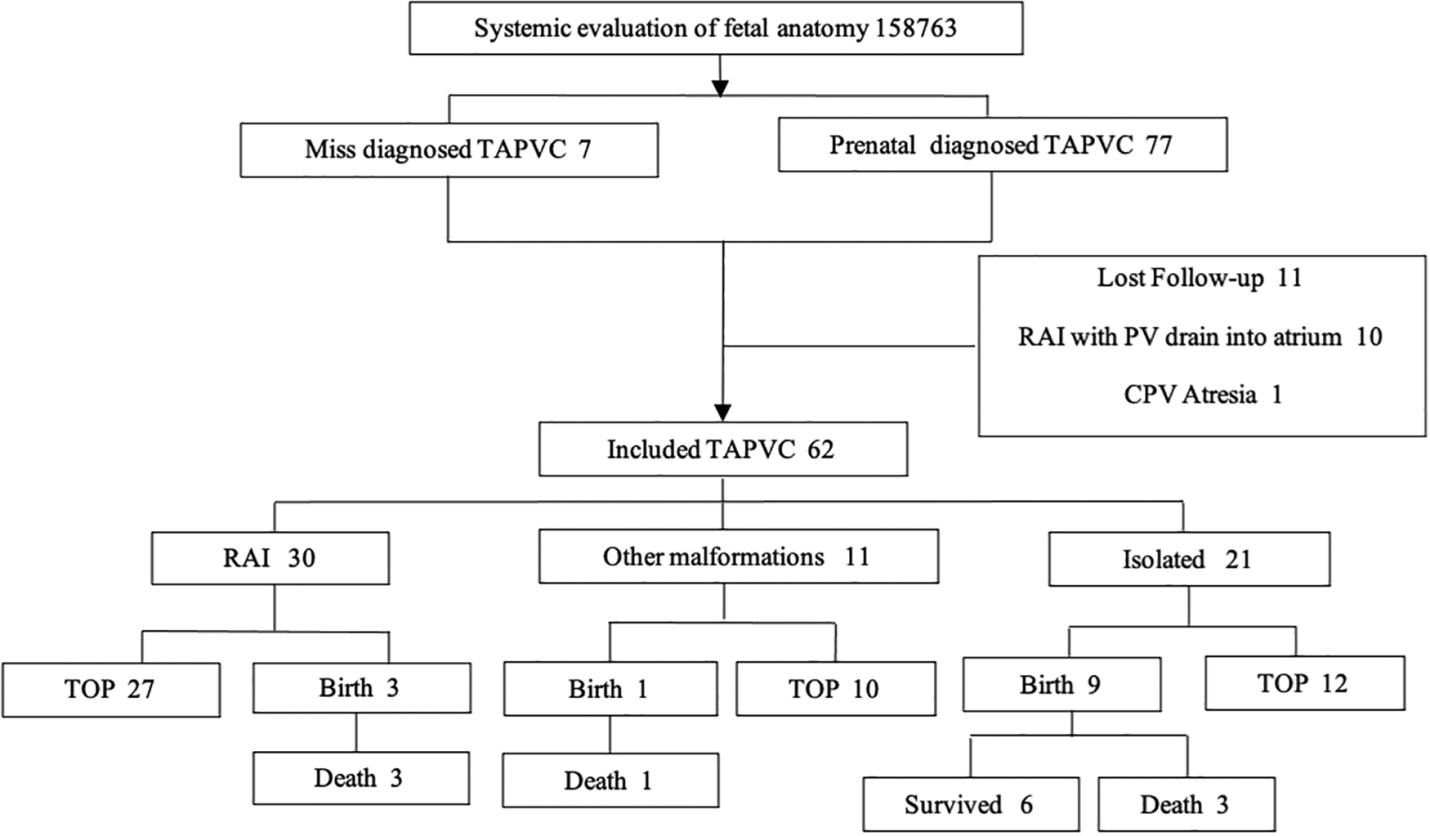
Flowchart of the enrollment in the study.

From June 2012 to June 2022, 158,763 pregnant women received prenatal ultrasonography and 84 cases of TAPVC were identified (5‰). Among them, the following cases were excluded from the study: 11 cases of loss to follow-up, 1 case of CPV atresia by postnatal autopsy, and 10 cases of RAI with PV entry into the atrium; 50 cases received TOP, of which 49 were confirmed as TAPVC by local pathological anatomy or combined casting of organs in thoracic and abdominal cavity; 6 cases chose to continue pregnancy and 7 were missed to be diagnosed prenatally. Live TAPVC infants suffered from cyanotic and respiratory distress, and the diagnosis was confirmed by echocardiography. Finally, 62 cases were included in the study (Figure 1). The mean age of the 62 pregnant women was 27.7 ± 3.5 years (19–36 years), and the mean gestational age (GA) was 25.56 ± 3.95 weeks (20.3–38 weeks). Of the 62 fetuses, 59 were singleton pregnancies and 3 were twin pregnancies (one of the fetuses was TAPVC). Among the 13 live TAPVC births, 4 infants died in the neonatal period without operation and 9 infants underwent surgery, including 5 total recoveries, 1 survival but with complication of SVC obstruction and collateral circulation, and 3 deaths after surgery.

### Classification and sonographic characterization of TAPVC

Of the 62 TAPVC cases, there were 20 supracardiac cases (32%), 12 intracardiac cases (19%), 21 infracardiac cases (34%), and 9 cases of mixed type (15%). Of the 20 cases of supracardiac TAPVC, CPV and single VV were found in 16 cases, of which 9 cases finally regurgitated into SVC, 4 into INN, 2 into AZV, 1 into left internal jugular vein (LIJV), and double VVs were reported in the remaining four cases, which regurgitated into SVC and INN (Table 1). Of the 12 cases with intracardiac TAPVC, 9 cases regurgitated into the RA through the CS and 3 cases drained directly into the RA (Table 2). Of the 21 cases of infracardiac TAPVC, 16 cases drained into the portal vein, 3 cases into ductus venous (DV), 1 into the hepatic vein (HV), and 1 into IVC (Table 3). Of the 9 cases of mixed TAPVC, 8 cases were combined with supracardiac and infracardiac drainage and 1 case was combined with supracardiac and intracardiac drainage (Table 4).

**Table 1:** Prenatal sonographic findings of supracardiac TAPVC.

** **	GA	Ventricle imbalance	Post-LA space enlarge	Absent PV-LA connection	CPV	3VV additional vessel	Prominent SVC	Site of connection	PVO	Associated malformation	Outcome	Postnatal diagnosis
1	25	Y	Y	Y	Y	Y	Y	CPV-VV-INN-SVC	Y	Isolated	TOP	Confirmed
2	25.5	N	Y	Y	Y	N	Y	CPV-SVC	N	Isolated	TOP	Confirmed
3	34.5	Y	Y	Y	Y	Y	Y	CPV-VV-SVC; LSPV-INN	Y	Isolated	TOP	Confirmed
4	24.6	N	Y	Y	Y	Y	Y	CPV-RVV-SVC; LIPV-LVV-INN	Y	Isolated	TOP	Confirmed
5	35.4	Y	Y	Y	Y	Y	Y	CPV-AZV-SVC	Y	Isolated	TOP	Confirmed
6	28	N	Y	Y	Y	Y	Y	CPV-VV-INN-SVC	N	Isolated	TOP	Confirmed
7	24	N	Y	N	Y	N	N	CPV-VV-INN-SVC	Y	Isolated	Live birth	Missed
8	27	Y	Y	Y	Y	Y	Y	CPV-VV-LSVC	N	RAI, AVSD, DORV, PS, BSVC	Live birth	Confirmed
9	24.3	Y	Y	Y	Y	Y	Y	CPV-VV-LSVC	Y	RAI, AVSD, DORV, PS, BSVC	TOP	Confirmed
10	22.5	Y	Y	Y	Y	Y	Y	CPV-VV-INN-SVC	N	RAI, DX, AVSD, PA, RAA, ASVR	TOP	Confirmed
11	23.5	N	Y	Y	Y	Y	Y	CPV-AZV-SVC	N	RAI, DX, AVSD, PA, RAA, AVSR	TOP	Confirmed
12	24	Y	Y	Y	Y	Y	Y	CPV-VV-SVC	N	RAI, AVSD, DORV, PS	TOP	Confirmed
13	23.6	Y	Y	Y	Y	Y	Y	LPV-CPV-VV-INN-SVC; RPV-SVC	N	RAI, AVSD, DORV, PA, RAA	TOP	Confirmed
14	23.5	N	Y	Y	Y	Y	Y	CPV-VV-SVC	N	RAI, SA, DOSV, PS, RAA	TOP	Confirmed
15	23.3	N	Y	Y	Y	Y	Y	CPV-VV-LIJV	N	RAI, SA, AVSD, DORV, PS, DLBCV	Live birth	Confirmed
16	23.4	N	Y	Y	Y	Y	Y	LCPV-VV-LSVC; RCPV-VV-RSVC	N	RAI, DOSV, PS, BSVC, PRUV	TOP	Confirmed
17	23.5	Y	Y	Y	Y	Y	Y	CPV-VV-SVC	N	RAI, MS, PTA, BSVC	TOP	Confirmed
18	24.2	Y	Y	Y	Y	Y	Y	CPV-VV-SVC	Y	RAI, DX, DORV, VSD, PA	TOP	Confirmed
19	24.3	N	Y	Y	Y	Y	Y	CPV-VV-SVC	N	RAI, LEVO, SA, SV, TGA, PS	TOP	Confirmed
20	23.6	N	Y	Y	Y	N	Y	CPV-SVC	N	DX, DOSV, AVSD, PS	TOP	Confirmed

TAPVC, total anomalous pulmonary venous connection; GA, gestational age; LA, left atrium; PV, pulmonary vein; CPV, common pulmonary vein; SVC, superior vena cava; PVO, pulmonary vein obstruction; VV, vertical vein; INN, innominate vein; TOP, termination of pregnancy; LSPV, left superior pulmonary vein; LIPV, left inferior pulmonary vein; AZV, azygos vein; LIJV, left internal jugular vein; RAI, right atrium isomerism; AVSD, atrioventricular septal defect; DORV, double outlet right ventricle; PS, pulmonary artery stenosis; PRUV, permanent right umbilical vein; VSD, interventricular septum; SV, single ventricle; TGA, transposition of great arteries; LSVC, left superior vena cave; PA, pulmonary atresia; 3VV, three vessel view; RVV, right vertical vein; LVV, left vertical vein; RPV, right pulmonary vein; LPV, left pulmonary vein; LCPV, left common pulmonary vein; RCPV, right pulmonary vein; RSVC, right superior vena cave; BSVC, bilateral superior vena cave; DX, dextrocardiac; RAA, right aortic arch; AVSR, abnormal systemic venous return; DOSV, double outlet single ventricle; DLBCV, dysplastic of left brachiocephalic vein; MS, mitral stenosis; LEVO, levocardia; SA, single atrium; PTA, persistent truncus arteriosus.

**Table 2:** Prenatal sonographic findings of intracardiac TAPVC.

** **	GA	Ventricle imbalance	Post-LA space enlarge	Absent of PV-LA connection	CPV	Dilated CS	Site of connection	PVO	Associated malformation	Outcome	Postnatal diagnosis
1	24	N	Y	Y	N	Y	PV-CS	N	Isolated	TOP	Confirmed
2	28	Y	Y	Y	Y	Y	CPV-CS	N	Isolated	Live birth	Confirmed
3	25.2	N	Y	Y	Y	N	CPV-RA	N	Isolated	TOP	Confirmed
4	23.2	Y	Y	Y	Y	Y	CPV-CS	N	Isolated	TOP	Confirmed
5	24.6	N	N	Y	Y	N	CPV-CS	N	Isolated	Live birth	Missed
6	24.5	N	N	Y	Y	N	CPV-CS	N	Isolated	Live birth	Missed
7	29.5	N	N	Y	Y	N	CPV-CS	N	Isolated	Live birth	Missed
8	26.6	Y	Y	Y	N	Y	PV-CS	N	LSVC	TOP	Confirmed
9	28.1	Y	Y	Y	Y	Y	CPV-CS	N	VSD, CoA	TOP	Confirmed
10	23.4	Y	Y	Y	Y	Y	CPV-RA	N	LSVC	TOP	Confirmed
11	20.3	N	Y	Y	Y	Y	CPV-CS	Y	DOSV, MA, PS, RAA	TOP	Confirmed
12	25.6	N	N	Y	N	N	PV-RA	N	DORV, VSD, PA, RAA, BSVC	TOP	Confirmed

TAPVC, total anomalous pulmonary venous connection; GA, gestational age; LA, left atrium; CPV, common pulmonary vein; CS, coronary sinus; PVO, pulmonary vein obstruction; PV, pulmonary vein; TOP, termination of pregnancy; RA, right atrium; VSD, interventricular septum; PS, pulmonary artery stenosis; DORV, double outlet right ventricle; LSVC, left superior vena cave; PA, pulmonary atresia; CoA, aortic coarctation; DOSV, double outlet single ventricle; MA, mitral atresia; RAA, right aortic arch; DORV, double outlet right ventricle; BSVC, bilateral superior vena cave.

**Table 3:** Prenatal sonographic findings of infracardiac TAPVC.

** **	GA	Ventricle imbalance	Post-LA space enlarge	Absent of PV-LA connection	CPV	Abdominal additional vessel	Site of connection	PVO	Associated malformation	Outcome	Postnatal diagnosis
1	22.2	Y	N	Y	Y	Y	CPV-VV-DV	Y	Isolated	TOP	Confirmed
2	24.1	N	Y	Y	Y	Y	CPV-VV-portal vein	N	Isolated	TOP	Confirmed
3	23.3	Y	Y	Y	Y	Y	CPV-VV-DV	Y	Isolated	Live birth	Confirmed
4	23	N	N	N	Y	N	CPV-VV-HV	Y	Isolated	Live birth	Missed
5	32.2	Y	Y	Y	Y	Y	CPV-VV-portal vein	Y	VSD, PA, oligoamnios	TOP	Confirmed
6	23.3	Y	Y	Y	Y	Y	CPV-VV-portal vein	Y	AVSD, PTA, BSVC	TOP	Confirmed
7	22	N	Y	N	Y	Y	CPV-VV-portal vein	N	TOF, holoprosencephaly	TOP	Confirmed
8	23.4	N	Y	Y	Y	Y	CPV-VV-DV	N	RAI, SV, ASD, PA	TOP	Confirmed
9	24.4	N	Y	Y	Y	Y	CPV-VV- portal vein	N	RAI, DX, AVSD, SV, TGA	TOP	Confirmed
10	25	Y	Y	Y	Y	Y	CPV-VV-PV	N	RAI, MA, VSD, DORV, PS, BSVC	TOP	Confirmed
11	23.4	Y	Y	Y	Y	Y	CPV-VV-portal vein	N	RAI, DX, AVSD, DORV, PA, BSVC	TOP	Confirmed
12	22.3	Y	Y	Y	Y	Y	CPV-VV- portal vein	N	RAI, AVSD, DORV, PS, RAA	TOP	Confirmed
13	28.5	N	Y	Y	Y	Y	CPV-VV-portal vein	N	RAI, AVSD, DOSV, PS, hiatal hernia	TOP	Confirmed
14	24.4	Y	Y	Y	Y	Y	CPV-VV-portal vein	N	RAI, AVSD, DORV, PS	TOP	Confirmed
15	22.4	N	Y	Y	Y	Y	CPV-VV-portal vein	N	RAI, SA, SV, PA	TOP	Confirmed
16	23.3	Y	Y	Y	Y	Y	CPV-VV-portal vein	N	RAI, DOSV, ASVR	TOP	Confirmed
17	23.4	Y	Y	Y	Y	Y	CPV-VV-portal vein	N	RAI, AVSD, DORV, PS, ASVR	TOP	Confirmed
18	22.2	N	Y	Y	Y	Y	CPV-VV-portal vein	N	RAI, AVSD, DOSV, PS, BSVC	TOP	Confirmed
19	23.4	Y	Y	Y	Y	Y	CPV-VV-portal vein	N	RAI, AVSD, DOSV, PS, BSVC, ASVR	TOP	Confirmed
20	24.1	Y	Y	Y	Y	Y	CPV-VV-portal vein	N	RAI, AVSD, DORV, PS, ASVR	TOP	Confirmed
21	38.2	N	Y	N	Y	Y	CPV-VV-IVC	N	RAI, SA, SV, CoA	TOP	Confirmed

TAPVC, total anomalous pulmonary venous connection; GA, gestational age; LA, left atrium; CPV, common pulmonary vein; PV, pulmonary vein; PVO, pulmonary vein obstruction; VV, vertical vein; DV, ductus venous; TOP, termination of pregnancy; HV, hepatic vein; VSD, interventricular septum; IVC, inferior vena cava; AVSD, atrioventricular septal defect; RAI, right atrium isomerism; SV, single ventricle; TGA, transposition of great arteries; DORV, double outlet right ventricle; TOF, tetralogy of fallot; SA, single atrium; PA, pulmonary atresia; PTA, persistent truncus arteriosus; BSVC, bilateral superior vena cave; ASD, atrium septal defect; DX, dextrocardiac; MA, mitral atresia; DORV, double outlet right ventricle; RAA, right aortic arch; AVSR, abnormal systemic venous return; DOSV, double outlet single ventricle; CoA, aortic coarctation.

**Table 4:** Prenatal sonographic findings of mixed TAPVC.

** **	GA	Ventricle imbalance	Post-LA space enlarge	Absent of PV-LA connection	CPV	Additional vessel	Site of connection	PVO	Associated malformation	Outcome	Postnatal diagnosis
1	22.2	Y	Y	Y	N	N	PV-CS; LSPV-VV-INN-SVC	Y	Isolated	Live birth	Modified
2	23.1	Y	Y	Y	Y	Y	CPV-VV-INN-SVC; LIPV-IVC	Y	Isolated	TOP	Confirmed
3	22.5	N	N	N	Y	N	CPV-VV- portal vein; LSPV-SVC	N	Isolated	Live birth	Missed
4	32.5	Y	Y	Y	Y	Y	CPV-VV-INN-SVC; CPV-VV-portal vein	N	TR (severe)	TOP	Confirmed
5	22.5	N	N	N	N	N	RPV-VV-SVC; LPV-VV-portal vein	N	RAA, SUA	Live birth	Missed
6	27.6	N	Y	Y	Y	Y	CPV-VV-portal vein; RSPV-SVC	N	RAI, DORV, VSD	Live birth	Confirmed
7	23.5	N	Y	Y	Y	Y	CPV-VV-INN-SVC; LIPV-VV-portal vein	Y	RAI, SA. DOSV, AVSD, PA, ASVR	TOP	Confirmed
8	31.1	Y	Y	Y	Y	Y	SCPV-VV-SVC; ICPV-VV-portal vein	N	RAI, AVSD, DORV, PS	TOP	Confirmed
9	22.6	Y	Y	Y	Y	Y	SCPV-SVC; ICPV-VV-DV	Y	RAI, AVSD, DORV, PA, RAA	TOP	Confirmed

TAPVC, total anomalous pulmonary venous connection; GA, gestational age; LA, left atrium; CPV, common pulmonary vein; PV, pulmonary vein; PVO, pulmonary vein obstruction; CS, coronary sinus; LSPV, left superior pulmonary vein; VV, vertical vein; INN, innominate vein; SVC, superior vena cava; TOP, termination of pregnancy; LIPV, left inferior pulmonary vein; IVC, inferior vena cava; RSPV, right superior pulmonary vein; LIPV, left inferior pulmonary vein; SUA, single umbilical artery; VSD, interventricular septum; AVSD, atrioventricular septal defect; PS, pulmonary artery stenosis; RAI, right atrium isomerism; SCPV, superior common pulmonary vein; ICPV, inferior common pulmonary vein; RPV, right pulmonary vein; LPV, left pulmonary vein; TR, tricuspid regurgitation; RAA, right aortic arch; DORV, double outlet right ventricle; DOSV, double outlet single ventricle; SA, single atrium; PA, pulmonary atresia; ASVR, abnormal systemic venous return.

**Figure 2:**
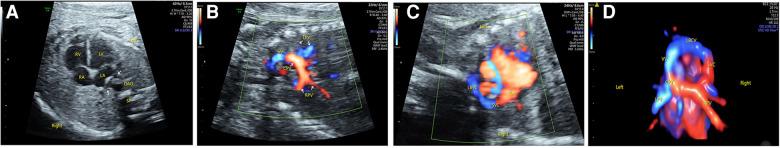
The prenatal sonographic features of supracardiac TAPVC. (**A**) The posterior wall of LA was smooth without orifice of PV, the pulmonary venous confluence behind the LA. (**B**) The left and right PVs converging to form a common PV truck, the CPV going upward as VV. (**C**) The VV drained into the SVC via the LBCV. (**D**) The entire drainage route of PVs. TAPVC, total anomalous pulmonary venous connection; PV, pulmonary vein; LA, left atrium; CPV, common pulmonary vein; VV, vertical vein; SVC, superior vena cava; LBCV, left brachiocephalic vein.

**Figure 3:**
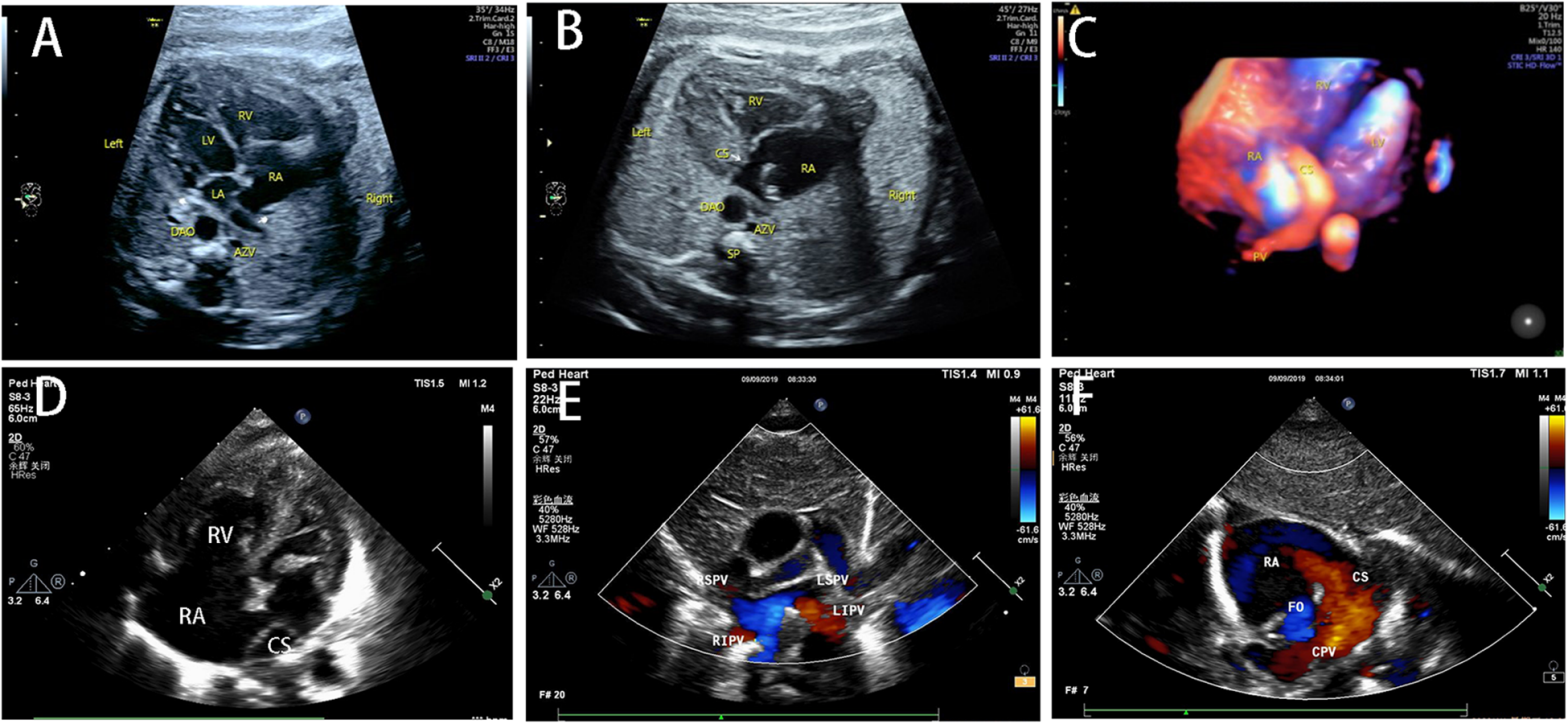
The prenatal and postnatal sonographic features of intracardiac TAPVC. (**A**) The four-chamber view showed ventricular discrepancy and no connection between PVs and LA. (**B**) The PVs drained into RA via dilated CS. (**C**) The entire drainage route of anomalous PVs. (**D**) The obvious asymmetry on the four-chamber view after birth. (**E**) All the PVs were not connected with LA. (**F**) The CPV drained into RA via dilated CS. TAPVC, total anomalous pulmonary venous connection; PV, pulmonary vein; LA, left atrium; RA, right atrium; CPV, common pulmonary vein; CS, coronary sinus.

**Figure 4:**
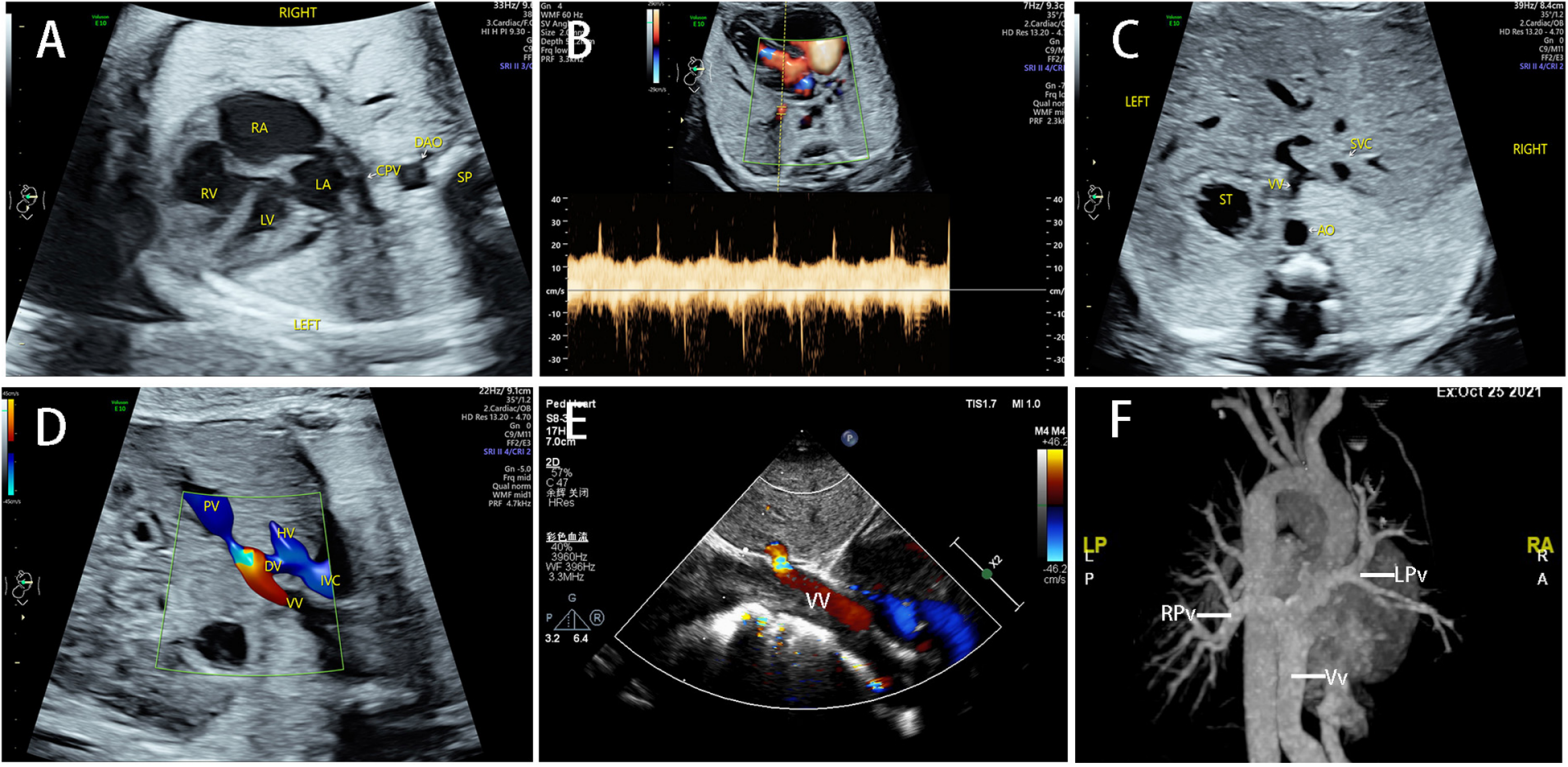
The sonographic features of infracardiac TAPVC and postnatal confirmation. (**A**) CPV was located behind the LA, with an increased posterior left atrium space. (**B**) The Doppler spectrum of PV was continuous instead of pulsatile. (**C**) The VV appeared as an extra vessel in the transabdominal section. (**D**) The VV drained into DV in a turbulent flow. (**E**) The obstruction was confirmed postnatally. (**F**) The CTA showed the entire drainage route of PVs. TAPVC, total anomalous pulmonary venous connection; PV, pulmonary vein; LA, left atrium; CPV, common pulmonary vein; VV, vertical vein; DV, ductus venous; CTA, computerized tomographic angiography.

**Figure 5:**
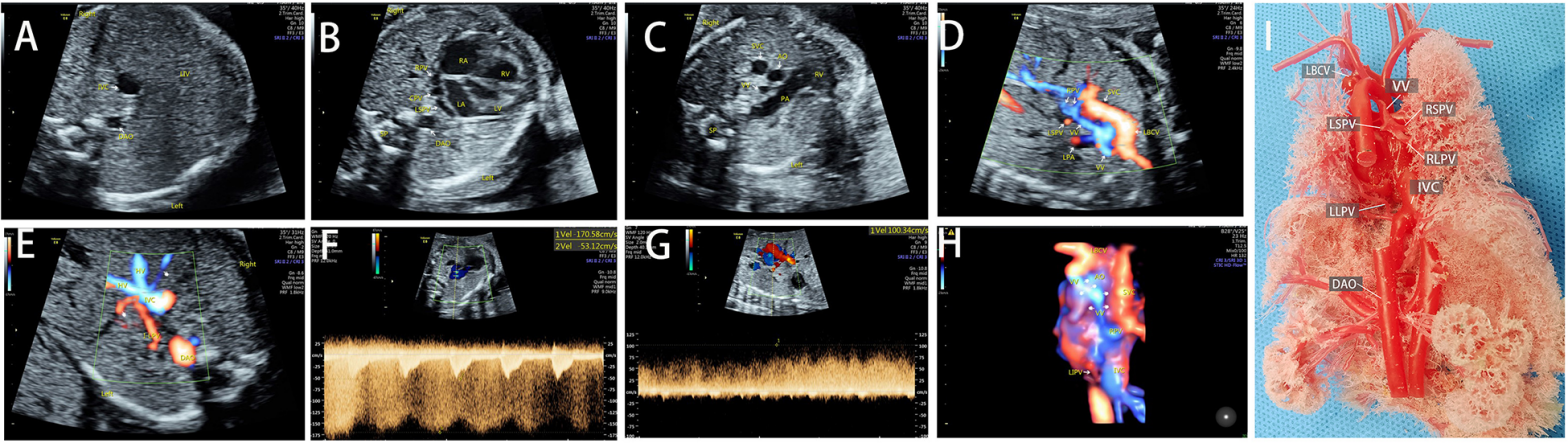
The sonographic features of mixed TAPVC and postnatal anatomic cardiovascular casting findings. (**A**) A dilated IVC in transabdominal section. (**B**) The ventricles were asymmetrical and the CPV was located behind the LA. (**C**) The VV appeared as an extra vessel behind the aorta. (**D**) The color Doppler showed the drainage of the RSPV, RLPV, and the LSPV into the LBCV via VV. (**E**) The LLPV drained into IVC. (**F,G**) Obstruction was confirmed in both the supracardiac and infracardiac pathways. (**H**) The entire drainage route of all PVs. (**I**): The postnatal casting of the TAPVC heart showed the RSPV, RLPV, and LSPV drainage into LBCV via the VV, and the LLPV drainage into the IVC. TAPVC, total anomalous pulmonary venous connection; IVC, inferior vena cava; CPV, common pulmonary vein; LA, left atrium; VV, vertical vein; RSPV, right superior pulmonary vein; RLPV, right low pulmonary vein; LSPV, left superior pulmonary vein; LBCV, left brachiocephalic vein; LLPV, left low pulmonary vein.

The common sonographic characteristics of TAPVC included small LA, smooth posterior wall of the LA, no PV orifice, increased distance between the LA and the descending aorta, and a continuous flow spectrum of the PV. CPV was shown in 56 cases, which appeared as an additional vessel behind or beside the LA. The sonographic image varied for different types of TAPVC. The supracardiac TAPVC featured an ascending VV, which was an additional vessel on a three-vessel trachea view and appeared as a centrifugal venous flow with dilated drainage site via color Doppler flow imaging (Figure 2). Echocardiographic findings of intracardiac TAPVC showed that the CPV either flew back into the RA through dilated CS or drained directly into the RA (Figure 3). The sonographic appearance of infracardiac TAPVC revealed a descending VV, an additional vessel in the transverse section of the abdomen, which appeared as a downward centrifugal vessel into the liver along with the IVC and the descending aorta in the sagittal section of the abdomen (Figure 4). Mixed TAPVC displayed multiple anomalous pulmonary venous drainage pathways, featuring two or more of the above ultrasonic findings (Figure 5).

### The obstruction of pulmonary veins in fetal TAPVC

Of the 62 cases of TAPVC, 17 cases were complicated with PVO, including 7 cases of supracardiac type (42%), 5 cases of infracardiac type (29%), 4 cases of mixed type (24%), and 1 case of intracardiac type (6%), with no statistical PVO difference among the different groups of TAPVC (*P* > 0.05) (Table 5). Among the seven supracardiac TAPVC cases, five cases reported PVO at the opening of the ascending VV into the SVC, one case at the opening of the ascending VV into the AZV, and one case at the VV crossing the bifurcation of the pulmonary artery. Of the five infracardiac TAPVC cases, the obstruction was located at the confluence of the descending VV into the DV in three cases and at the confluence of the descending VV into the PV in two cases. In the intracardiac TAPVC case, four PVs formed a CPV and the VV were tortuous, in which the obstruction was located at the bifurcation of pulmonary artery and confluent to the CS. Of the four cases of mixed TAPVC, three cases reported a mixture of supracardiac and infracardiac TAPVC, in which the obstruction occurred during the infracardiac drainage when the descending VV entered the IVC, portal vein, and DV, and one case displaying obstruction in the supracardiac route, with the ascending VV crossing the left pulmonary artery. The remaining case displayed a mixture of intracardiac and supracardiac TAPVC, in which the obstruction was found in the supracardiac and intracardiac pathways, respectively, at the point where the left inferior pulmonary vein (LIPV) joined the CPV and at the point where the VV joined the INN. All the PVO cases displayed the sonographic features of the tortuous and slender obstructive site, with turbulent blood flow by color Doppler imaging and continuous high-speed blood flow by spectrum Doppler imaging at a maximum velocity of 110 ± 33 cm/s (58–170 cm/s).

**Table 5:** The drainage route and obstruction of TAPVC.

Classification	Drainage route (*n*)	PVO*
Supracardiac	CPV-VV-RSVC (7)	1
	Bilateral VV (4)	2
	CPV-VV-LBCV-RSVC (4)	2
	CPV-VV-LSVC (2)	1
	CPV-AZV-SVC (2)	1
	CPV-VV-LIJV (1)	0
Total	20/62	7/20
Intracardiac	CPV-CS (7)	1
	PV-CS (2)	0
	CPV-RA (2)	0
	PV-RA (1)	0
Total	12/62	1/12
Infracardiac	CPV-VV-portal vein (16)	2
	CPV-VV-DV (3)	2
	CPV-VV-IVC (1)	1
	CPV-VV-HV (1)	0
Total	21/62	5/21
Mixed type (9/62)	CPV-SVC and PV-VV-portal vein (6)	1
	SCPV-VV-SVC and ICPV-VV-DV (1)	1
	CPV-VV-SVC and LIPV- IVC (1)	1
	LSPV-VV-INN-SVC and PV-CS (1)	1
Total	9/62	4/9

TAPVC, total anomalous pulmonary venous connection; PVO, pulmonary vein obstruction; CPV, common pulmonary vein; PV, pulmonary vein; VV, vertical vein; LBCV, left brachiocephalic vein; SVC, superior vena cava; LIJV, left internal jugular vein; CS, coronary sinus; RA, right atrium; DV, ductus venous; IVC, inferior vena cava; HV, hepatic vein; SCPV, superior common pulmonary vein; ICPV, inferior common pulmonary vein; INN, innominate vein; RSVC, right superior vena cave; LSVC, left superior vena cave.

* *P*-value was obtained from chi-square test with continuity correction. The difference between PVO in four groups is not statistical significance (*P *= 0.142).

### The associated malformations of the fetal TAPVC

**Figure 6:**
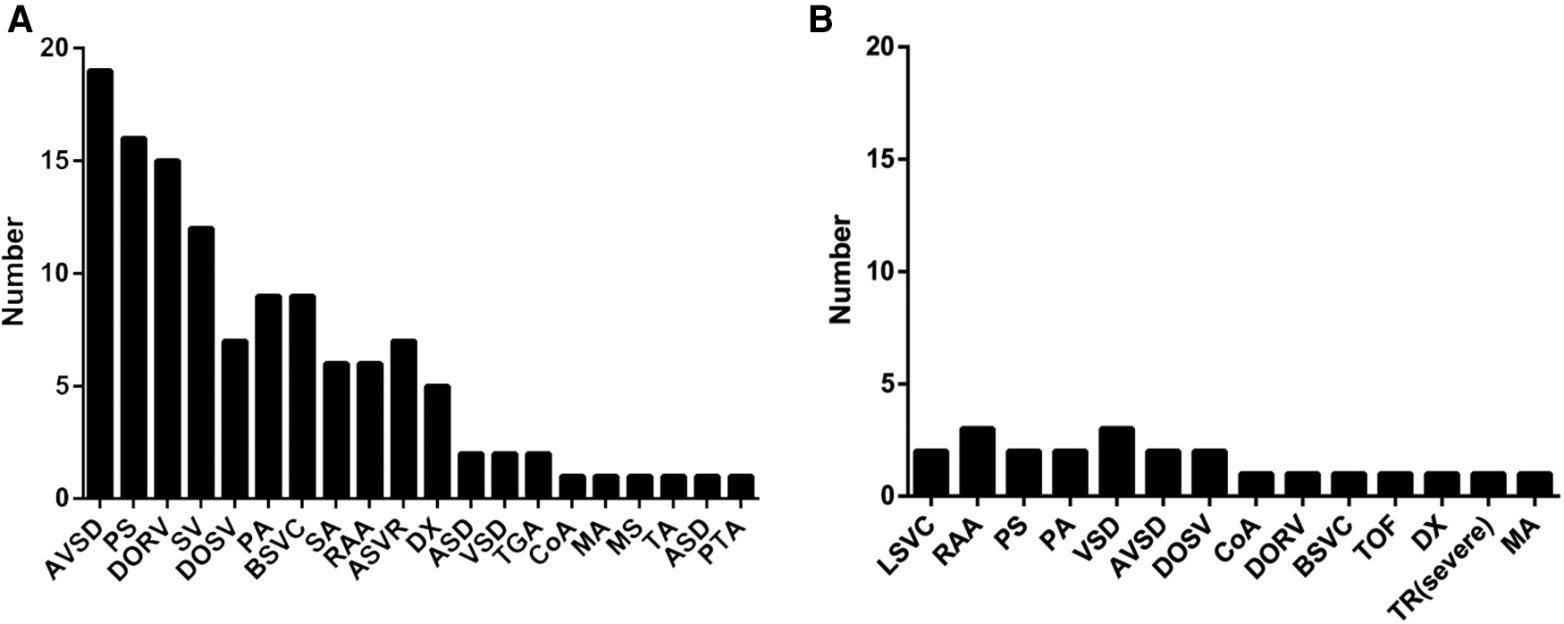
Obvious differences of the associated intracardiac malformations between TAPVC with RAI (**A**) and without RAI (**B**). TAPVC with RAI is usually associated with a series of complex intracardiac anomalies and TAPVC without RAI can be isolated or accompanied with other anomalies. TAPVC, total anomalous pulmonary venous connection; right atrium isomerism.

Of the 62 TAPVC cases, 41 cases were complicated with other intracardiac and extracardiac malformations, including 30 RAI cases and 11 cases with other intracardiac and extracardiac malformations, and the remaining 21 cases were isolated TAPVC. In the 30 TAPVC cases combined with RAI, the common concomitant malformations included atrioventricular septal defect (AVSD) (19/30), pulmonary artery stenosis (PS) (16/30), double outlet right ventricle (DORV) (15/30), single ventricle (SV) (12/30), etc., and the rare complicated malformations were interventricular septum (VSD) (2/30), transposition of great arteries (TGA) (2/30), etc. (Figure 6A). In the 11 TAPVC cases complicated with other cardiac malformations, common concomitant malformations were VSD (3/11), right aortic arch (3/11), left superior vena cave (LSVC) (2/11), PS (2/11), etc. (Figure 6B). TAPVC cases combined with extracardiac malformations displayed cleft lip and palate, holoprosencephaly, hiatal hernia, oligohydramnios, and permanent right umbilical vein (PRUV), one case per category, and single umbilical artery (SUA) in two cases. Of the 62 TAPVC cases, seven cases underwent chromosome tests, which reported chromosome normality in six cases and inversion of chromosome 9 in one case.

### The diagnostic accuracy of the four-step prenatal ultrasonography

Of the 62 cases of TAPVC, the diagnostic accuracy of the four different TAPVC types by the four-step prenatal ultrasonography was 95%, 75%, 95%, and 77%, respectively, with no statistical significance (*P* > 0.05) (Table 6). Thirty cases complicated with RAI and 10 cases combined with other intracardiac and extracardiac abnormalities were correctly diagnosed by the proposed prenatal ultrasonic procedure, while 1 case was missed, which was a mixed type (supracardiac and infracardiac) with right aortic arch and SUA. Of the 21 isolated cases of TAPVC, 14 cases were correctly diagnosed; 1 case was diagnosed as intracardiac type prenatally, which was postnatally confirmed to be a mixed type (supracardiac and intracardiac) with obstruction of the supracardiac and intracardiac drainage pathway by echocardiography; 6 cases were missed. As shown in Table 6, the proposed four-step prenatal ultrasonography reported a higher accuracy in diagnosing TAPVC cases that were complicated with RAI (*P* < 0.05).

**Table 6:** Comparison of the diagnostic rate of different types of TAPVC and comparison of the diagnostic rate of TAPVC complicated with other malformations.

Classification	Correct (*n*)	Incorrect (*n*)	*P* *
Supracardiac	19	1	0.195
Intracardiac	9	3	
Infracardiac	20	1	
Mixed	7	2	
Complicated with RAI	30	0	0.003
Other anomalies	10	1	
Isolated	15	6	

TAPVC, total anomalous pulmonary venous connection; RAI, right atrium isomerism.

The difference of diagnostic rate between four TAPVC groups is not statistically significant (*P *= 0.195). The difference of diagnostic rate between TAPVC complicated with other malformations is statistically significant (*P *= 0.003).

* *P*-value was obtained from chi-square test with continuity correction.

## Discussion

Prenatal diagnosis of TAPVC has always been a great challenge, especially in isolated cases (16). Due to its low incidence and difficulty in obtaining detailed images, it is rarely detected during obstetric screening in the second trimester of pregnancy (17). Diagnostic accuracy has been improved thanks to sonographic advances and experience accumulation in the past decade. Several retrospective studies have documented many suspicious signs of TAPVC during prenatal ultrasound screening, including a small LA, lengthened distance between the LA and the descending aorta, the smooth posterior wall of the LA, obscured orifices of the PV, additional vessels in some sections, centrifugal venous flow, and dilatated fetal systemic veins (e.g., SVC, INN, AZV, IVC, or CS) (15). When the above-mentioned suspicious TAPVC diagnostic clues appear, the use of prenatal ultrasound four-step observation can help improve the diagnosis and classification of TAPVC, especially when TAPVC is associated with other malformations. In this article, we found that after postpartum validation or pathological comparison, the prenatal correct diagnosis rates of the supracardiac, intracardiac, infracardiac, and mixed types were 95%, 75%, 95%, and 77%, respectively. Thirty TAPVC cases with RAI were accurately detected by the proposed prenatal ultrasonography, though one case of TAPVC with other extracardiac and intracardiac malformations was missed. Six cases of isolated TAPVC were missed. The potential explanation for missed diagnosis may lie in the following considerations: (1) The entry of fetal PV into the LA is falsely identified during the examination, since the echoes of bronchial and pulmonary arteries may interfere with the orifices of the PVs at the top of the LA and may be mistaken for the connection with the LA; (2) Due to the small size of the fetal PV, the fetal position, and the thickness of abdominal wall of the pregnant woman, two-dimensional ultrasonography may not clearly delineate the lumen of the PV; (3) The dilated systemic veins and VV in the drainage site were overlooked because of inexperience. These weaknesses may be remedied by adjusting the blood flow velocity scale to about 20 cm/s during color Doppler flow imaging and verifying the connection by spectrum Doppler imaging. In addition, an extra vessel in the trachea section or abdominal cross-section is an important indicator for TAPVC diagnosis.

Of the four types of TAPVC, the infracardiac and mixed types of TAPVC have a poor prognosis (18). Even for the same type, the prognosis differs for different drainage paths. For example, for different subtypes of supracardiac TAPVC, direct access to the right SVC via the right VV is more likely to result in postoperative PVO (19). The prognosis of intracardiac TAPVC is generally considered good (20). In this study, four cases of isolated intracardiac TAPVC had a good prognosis after surgical treatment. However, the prognosis of intracardiac TAPVC varies for different sites of connection. Studies have shown that the number of PV orifices, the length of the drainage path, and the drainage through the roof of the RA are associated with postoperative PVO (21). Therefore, the prenatal diagnosis of TAPVC should delineate not only the type of TAPVC but also the specific connection of ectopic PV and the length of the drainage path.

PVO is also an essential factor for a poor prognosis, and preoperative PVO is closely associated with mortality (18, 22). The available literature indicates that preoperative PVO occurs in approximately 25%–50% of cases (23, 24) and the incidence of PVO in prenatal TAPVC fetuses is as high as 34.1% (95% CI: 22.7%–47.7%) (3), signifying that one-third of the cases are likely to show evidence of prenatal obstruction. Our cohort found PVO in 17 cases (17/62, 27.4%): prenatally in 14 cases and postnatally in 3 cases. The disparity may be attributed to the following facts: as, in the current study, the average GA for the prenatal diagnosis was 25.56 ± 3.95 weeks (20.3–38 weeks) and most cases were diagnosed in the second trimester, the minor pulmonary blood flow may result in unnoticeable or no obstruction while with the progression of gestation, the increase in pulmonary blood flow may induce the obstruction; most cases in the current study received induced labor after TAPVC diagnosis and no follow-up was conducted in late pregnancy. Therefore, fewer PVO cases were diagnosed in this cohort.

PV stenosis is often caused by insertion site stenosis and external organ compression, and the longer the drainage path is, the more likely it is to be obstructed. PVO can occur at any site and aggravate during pregnancy, especially when pulmonary vascular resistance decreases, leading to increased blood flow (25). PVO can also lead to PV remodeling and even progress to atresia (26). Studies have suggested that an earlier occurrence of PVO may indicate a poorer prognosis, as it will lead to pulmonary vascular remodeling and may even compromise the efficacy of a successful surgery (26). In this study, of the 13 live births, 4 PVO infants died in the neonatal period and the prognosis of fetuses without PVO was good. Therefore, PVO should rank as an essential part of prenatal TAPVC evaluation.

TAPVC can be combined with a series of intracardiac and extracardiac malformations. The complication with RAI, one of the most common malformations in TAPVC, is associated with a worse prognosis due to faster disease progression and a greater chance of multi-operation after repair of the PV (27). In our cohort, 30 cases (30/62, 48%) were complicated with RAI. RAI is characterized by complex intracardiac abnormalities, such as incongruent visceral atria position, complete AVSD, DORV, and right ventricular outflow tract obstruction (28). Due to the bilateral right atrial structure, evaluation of pulmonary venous return is an important step in diagnosing RAI (29, 30). In addition to RAI, TAPVC may be associated with other extracardiac and intracardiac malformations, such as TGA, tetralogy of fallot (TOF), single atrium (SA), persistent truncus arteriosus (PTA), tricuspid atresia (TA), hypoplastic left heart syndrome, pulmonary atresia (PA), VSD, aortic coarctation (CoA), and other abnormalities (25). In this cohort, 11 cases were complicated with other intracardiac or extracardiac malformations. The associated anomalies, especially complex intracardiac anomalies that require secondary surgery, are also associated with a poor prognosis of TAPVC. Some deformations require no remedy or can be simultaneously corrected during the repair of the PV, in which the deformation may not be a risk factor for postoperative death (5).

The mode of inheritance of TAPVC remains unknown. However, it may be a single gene inheritance according to the existent literature (25). In the affected families, the abnormal pulmonary venous connection is a common characteristic, but the location of the abnormal connection is inconsistent (31). TAPVC is associated with several syndromes, such as asplenia, polysplenia, and Cat's eye syndrome (25). In the current study, the chromosome examination of seven cases revealed chromosome 9 inversion in one case and no abnormality in the other six cases.

### Limitation

Several limitations should be noted in the current study. First, as a retrospective study, the archived images may affect the quality and integrity of data sources. Second, the sample size is quite small. In this study, only 13 TAPVC fetuses were retrieved for analysis, which was unfavorable for evaluating the prognosis of TAPVC. Third, the accuracy of the proposed four-step prenatal ultrasonography may be compromised by the complexity of the enrolled cases, in which the RAI complication and isolated TAPVC may confound the results of the analysis. Finally, a more detailed spectrum analysis of the VV and anomalous PV should be provided.

## Conclusions

The proposed four-step ultrasonography can satisfactorily delineate fetal TAPVC classification, the presence of PVO, and intracardiac and extracardiac abnormalities, which offers beneficial support for prenatal counseling and prognostic improvement of postnatal TAPVC. The retrospective analysis also reveals that isolated TAPVC is more prone to be missed in diagnosis.

## Data Availability

The original contributions presented in the study are included in the article, further inquiries can be directed to the corresponding authors.
